# Discovering the ecological structure of different macrophyte groups in rivers using non-parametric and parametric multivariate ordination techniques

**DOI:** 10.1038/s41598-024-64089-2

**Published:** 2024-06-10

**Authors:** Anna Budka, Krzysztof Szoszkiewicz, Karol Pietruczuk, Tropikë Agaj

**Affiliations:** 1 Department of Construction and Geoengineering, Poznan University of Life Sciences, Wojska Polskiego 28, 60-637 Poznan, Poland; 2 Department of Ecology and Environmental Protection, Poznan University of Life Sciences, Wojska Polskiego 28, 60-637 Poznan, Poland; 3 BIOLEKO Badania i Dokumentacja Środowiskowa, Klasztorna 8, 62-010 Pobiedziska, Poland

**Keywords:** Ordination methods, Site classification, Habitat gradient, Water–vegetation, Ecology, Environmental sciences, Hydrology

## Abstract

This paper analyses various methods of ecological ordering that are often used in modelling the relationship between vegetation and habitat. The results of direct gradient ordination by Canonical correspondence analysis (CCA), which is based on correlation, were compared with Non-metric multidimensional scaling (NMDS), which is based on rank analyses. Both tools were also compared with Detrended correspondence analysis (DCA), which is a popular indirect gradient analysis method. The macrophyte assessment was conducted at 98 river locations in the lowland regions of Poland. Each of the surveyed locations falls within a consistent abiotic category: small to medium-sized lowland rivers with a sandy bottom. Habitat elements analysed included limnological variables and geographic parameters, and the botanical survey focused on submerged macrophytes, including vascular plants, as well as bryophytes and algae. Firstly, it was shown that various analytical tools for determining the importance of ecological factors (Monte Carlo test, BIOENV) identify slightly different significant factors responsible for the development of macrophytes in rivers. Secondly, considerable similarity was found in the structure of macrophyte communities generated on NMDS and DCA biplots, while macrophyte communities were presented very differently based on CCA. Thirdly, the ecological preferences of aquatic plants based on one-dimensional analyses primarily reflected the results of CCA, whereas they did not always follow the ecological pattern revealed by NMDS. Finally, by conducting separate studies for non-vascular plants and vascular macrophytes, it was confirmed that different ecological drivers are responsible for the development of particular groups of macrophytes

## Introduction

The spatial variation of species distribution is a prominent characteristic observed in aquatic ecosystems. This variability is influenced by a multitude of biological, physical, and chemical properties that contribute to the differentiation of species composition, functional groups, and communities^1–3^. Numerous studies conducted in aquatic environments have examined the interplay between the structure of biological communities and environmental factors. However, these relationships have proven to be highly complex, posing a challenge for analysis^1,4,5^. Notably, several of these studies have focused on aquatic plants, specifically investigating the ecological differences among various macrophyte groups^3,6^.

Aquatic macrophytes are essential components of river ecosystems involved in energy flow and biogeochemical cycling, providing habitat and serving as a food source for many organisms^7–9^. This is a diverse group of aquatic photosynthetic organisms, all large enough to see with the naked eye^10^. Macrophytes comprise a variety of taxonomic groups, from vascular aquatic plants (seed-bearing plants and ferns) to various non-vascular plants such as bryophytes (mosses and liverworts) and structural macroalgae^8,10^. Macrophytes exhibit various life forms—floating, submerged, and emergent—based on their placement within the water column. This functional trait is significant and can be affected by abiotic factors^8,9^.

The development of macrophyte assemblages strongly depends on various abiotic and biotic factors. It is assumed that the most important of them are nutrient concentrations^10–13^, flow velocity^10,14,15^, hydrological conditions^10,16,17^, hydromorphological modifications^18,19^, and landscape pattern^20–22^. A precise analysis of the ecological dependencies of macrophytes is fundamental, because these organisms are widely used in the biomonitoring of water quality^23–25^. Moreover, knowledge about habitat requirements is essential to formulate appropriate measures for conservation preservation and for the restoration of valuable macrophyte vegetation types^26,27^.

The structure of plant assemblages can be analysed using various ordination techniques enabling the ordering of species or sites with respect to environmental gradients^28^. The information on ecological matrices can be reduced to a few significant axes of environmental variation. Ordination is a term that describes this process of reducing the information needed to represent a matrix. The term derives from the concept of ordinating or putting things in order. With ordination approaches, we attempt to take a high-dimensional data matrix and explain its patterns with a small number of axes. Although this is a general concept across scientific disciplines, the term ordination is generally used only in ecology^26,27,29^.

Multivariate methods are particularly frequently used for ordination. They include direct gradient analysis techniques such as canonical correspondence analysis (CCA^30^) and indirect gradient analysis techniques such as non-metric multidimensional scaling (NMDS^29^) and detrended correspondence analysis (DCA^31^). Many authors have put forward advantages and disadvantages of these approaches in determining the ecological relationships that differentiate species distribution, although precision and caution in their interpretation are always recommended^31^. The use of these algorithms in practice allows the initial ordering of data (both habitat and biological) according to the gradient of the most important factor differentiating the variability on the research plots. This procedure allows one to capture as many differences as possible between individual surfaces in the system of the first two coordinate axes.

As a method of direct gradient analysis, CCA ordinates the species data largely according to habitat variation^30,32^. The method extracts synthetic gradients from environmental data sets, which are the basis for visualising the different habitat preferences of taxa on an ordinal diagram. In CCA, the species data are initially chi-square transformed, but their relationship with the environmental variables is based on the linear correlation model using a mutual averaging algorithm.

NMDS is a method of indirect gradient analysis of community data based on a ranging algorithm according to similarity in species composition. The measure of species similarity of research plots used in this scaling is the Jaccard distance matrix^33,34^. Moreover, the relationship of the species data with environmental gradients can be investigated to some extent in a subsequent step by correlating the ordination scores with the environmental variables. This involves the use of a simple optimisation procedure in R: Best Subset of Environmental Variables with Maximum (Rank) Correlation with Community Dissimilarities (BIOENV^34^), which selects the best subset of environmental variables, maximising the (modified) rank correlation between the biotic and abiotic similarity matrices so that the Euclidean distances of the scaled environmental variables have a maximum (ranking) correlation with different species of organisms belonging to independent groups^34^.

DCA is another indirect gradient analysis method based on the mutual averaging algorithm. It improves multidimensional-rescaling ordinations by reducing the arch effect produced by other ordination techniques^31,35^. The distances among samples (sites) in the reduced ordination space approximate chi-square distances among samples in the full-dimensional space; any object found near the point representing a species is likely to contain a high contribution of that species. Sample scores are calculated as the means of species scores occurring in the sample, weighted by species abundances. Usually, non-biased compliance analysis (DCA) is used for the initial diagnosis of community data, often accompanied by CCA as a method of showing vegetation–habitat relationships. In many situations, information delivered by the DCA axis can indicate ecological gradients, which is especially helpful when lacking abiotic information, and when obtaining such data is expensive and time-consuming^30^.

This study aimed to compare different ordination techniques to examine their distinct perspectives on the relationship between macrophytes and environmental characteristics of the rivers in Poland, encompassing physical and chemical parameters along with geographical indicators. The ecological requirenments were tested on on both non-vascular plants and vascular macrophytes. The direct gradient ordination method CCA, based on correlation, was compared with the rank-based NMDS, while both were further compared with the popular indirect gradient analysis DCA. The observed relationships were compared with reference to basic one-dimensional statistics describing the distribution of environmental variables. The comparisons were completed separately for two plant groups: vascular macrophytes and non-vascular plants, which encompass bryophytes and structural algae.

## Results

### Taxonomic identification

The study considered macrophytes occurring in rivers, and the analyses were carried out separately for two groups: non-vascular taxa (algae and bryophytes) and vascular macrophytes (seed-bearing plants and ferns). Non-vascular macrophytes were identified at 55 sites, with a total of 7 taxa, two of which are bryophytes (*Fontinalis antipyretica* and *Leptodictyum riparium*), and the rest are structural algae (*Cladophora* sp., *Oedogonium* sp., *Rhizoclonium* sp*.*, *Spirogyra* sp*.*, *Vaucheria* sp.). Vascular macrophytes were recorded at 98 sites, where 24 taxa were identified (Table 1).

**Table 1:** List of taxa identified in the studied rivers.

Non-vascular macrophytes	Abbreviation	Vascular macrophytes (cont.)	Abbrievation
*Cladophora* sp.	*Cla sp*	*Potamogeton crispus* L.	*Pot cri*
*Fontinalis antipyretica* Hedw.	*Fon ant*	*Potamogeton lucens* L.	*Pot luc*
*Leptodictyum riparium* Hedw. Warnst	*Lep rip*	*Potamogeton natans *L.	*Pot nat*
*Oedogonium* sp.	*Oed sp*	*Potamogeton nodosus* Poir.	*Pot nod*
*Rhizoclonium* sp.	*Rhi sp*	*Potamogeton pectinatus* L.	*Pot pec*
*Spirogyra* sp.	*Spi sp*	*Potamogeton perfoliatus* L.	*Pot per*
*Vaucheria* sp.	*Vauch sp*	*Potamogeton praelongus* Wulfen.	*Pot pra*
Vascular macrophytes	Abbreviation	*Ranunculus aquatilis* (L.) Dumort.	*Ran aqu*
*Berula erecta* L.	*Beru ere*	*Ranunculus circinatus* (Sibth.) Fr.	*Ran cir*
*Callitriche* sp.	*Cal sp*	*Ranunculus fluitans* (Lam.) Wimm.	*Ran flu*
*Ceratophyllum demersum* L. s. s	*Cer dem*	*Ranunculus trichophyllus* (Chaix) Bosch.	*Ran tri*
*Ceratophyllum submersum* L.	*Cer sub*	*Sagittaria sagittifolia* L.	*Sag sag*
*Elodea canadensis* Michx.	*Elo can*	*Scirpus lacustris* (L.) Palla.	*Sci lac*
*Myriophyllum spicatum* L.	*Myr spi*	*Sium latifolium* L.	*Siu lat*
*Nuphar lutea* Sm.	*Nup lut*	*Sparganium emersum* Rehmann.	*Spa em*
*Potamogeton compressus* L.	*Pot com*	*Stratiotes aloides* L.	*Str alo*

An initial characterisation of the ecological properties of the identified macrophyte groups is given in Table 2. A wide nutrient gradient was found between the studied rivers, especially for ammonium (the variation coefficient was 1.36 for non-vascular macrophytes and as high as 1.53 for vascular plants). On the other hand, the pH level was relatively very stable. Most of the variables meet normal distribution criteria: low skewness and correspondence between the mean and median. Slightly increased skewness was observed only for ammonium (3.17 for non-vascular and 3.33 for vascular macrophytes) and organic-P (2.3 and 2.7 respectively).

**Table 2:** Descriptive statistics of environmental variables for sites where (a) non-vascular macrophytes and (b) vascular macrophytes were recorded.

Parameters	Latitude	Longitude	Ammonium	Nitrate	Organic-N	BOD-5	Ortho-P	Organic-P	pH	Electrical conductivity
Variable	Geographical coordinates	Geographical coordinates	${\text{mg N - NH}}_{{4}} {\text{/I}}$	${\text{mg N - NH}}_{{3}} {\text{/I}}$	${\text{mg N/I}}$	${\text{mg O}}_{{2}} {\text{/I}}$	${\text{mg PO}}_{{4}} {\text{/I}}$	${\text{mg P/I}}$	pH scale	S/cm
Non-vascular macrophytes										
Mean	472,395	534,090	0.538	2.784	1.37	3.23	0.553	0.335	7.82	627.9
Median	413,498	508,185	0.365	2.147	1.26	2.93	0.493	0.287	7.87	626.2
Standard deviation	155,801	87,473	0.729	2.615	0.59	1.28	0.419	0.255	0.22	217.5
Coefficient of variation	0.30	0.20	1.36	0.94	0.43	0.40	0.76	0.76	0.03	0.35
Minimum	206,825	413,056	0.013	0.08	0.46	1.48	0.03	0.07	7.36	249.5
Maximum	787,083	725,554	4.091	12.49	3.15	6.51	1.91	1.45	8.16	1095.3
Skewness	0.50	0.70	3.17	1.84	1.10	0.83	1.37	2.30	− 0.52	0.22
Vascularmacrophytes										
Mean	477,013	562,303	0.485	2.294	1.25	3.06	0.454	0.299	7.82	567.4
Median	404,820	545,787	0.241	1.675	1.13	2.82	0.324	0.237	7.86	533.8
Standard deviation	169,015	102,595	0.739	2.338	0.59	1.22	0.397	0.254	0.22	219.9
Coefficient of variation	0.40	0.20	1.53	1.02	0.47	0.40	0.88	0.85	0.03	0.39
Minimum	206,825	407,411	0.013	0.08	0.45	1.09	0.03	0.04	7.21	236.0
Maximum	827,241	756,525	4.212	12.49	3.15	6.51	1.91	1.53	8.34	1095.3
Skewness	0.50	0.40	3.33	2.05	1.32	0.92	1.64	2.70	− 0.53	0.55

### Selection of significant environmental variables

Based on the Monte Carlo test, an analysis of the influence of habitat features on the vegetation ordering model was performed, and significant environmental variables were distinguished for the CCA analysis (p < 0.05) (Table 3). For non-vascular plants, electrical conductivity and latitude were identified as significant variables, while for vascular macrophytes, the significant variables were BOD, latitude, and pH.

**Table 3:** Environment variables with the best correlation with plant community data based on the Monte Carlo method, permutation tests for the purpose of CCA.

Macrophyte group	Variable	*F*-ratio	p-value	Variance explained	V.e. all var
Non-vascular macrophytes	Electrical conductivity	3.78	0.002	0.23	0.70
	Latitude	2.22	0.038	0.09	
Vascular macrophytes	BOD	4.56	0.002	0.18	0.73
	Latitude	3.05	0.002	0.14	
	pH	2.42	0.002	0.09	

Based on the BIOENV function, the best subset of environmental variables for the NMDS analysis was identified (Table 4), which for non-vascular macrophytes included parameters such as electrical conductivity and pH (correlation = 0.1417), while for vascular vascular macrophytes significance was found for latitude, ammonium, BOD, phosphate, and pH (correlation = 0.1827). The correlation between the two matrices was significant in both cases (p < 0.05), which indicated a strong relationship between the structure of macrophyte communities and selected environmental variables. In addition, Table 4 includes R^2^ (coefficient of determination)—this is a measure of the "fit" of the NMDS model to the data and its significance, reflecting correlation between the environmental (external) variable and the results of the ordinance projected onto the considered variables (not onto the axes). P-value in the of NMDS is typically associated assess the significance of relationships between the data and clustering in the NMDS space.

**Table 4:** Non-metric multidimensional scaling (NMDS).

Macrophyte group	Variable	R^2^	*p*-value	stress
Non-vascular macrophytes	Electrical conductivity	0.0595	0.197	0.02001
	pH	0.1267	0.033	
Vascular macrophytes	Latitude	0.0558	0.059	0.1425
	Ammonium	0.1263	0.001	
	BOD-5	0.1216	0.003	
	Ortho-P	0.0725	0.028	
	pH	0.0021	0.914	

The significance of environmental variables was determined by permutation tests in fitted NMDS ordination for environmental variables with the best correlation with community data based on the BIOENV function.

Selecting variables based on BIOENV results before proceeding with NMDS analysis is a strategic approach that maximizes the accuracy and effectiveness of the analysis.

### Univariate analysis

**Figure 1:**
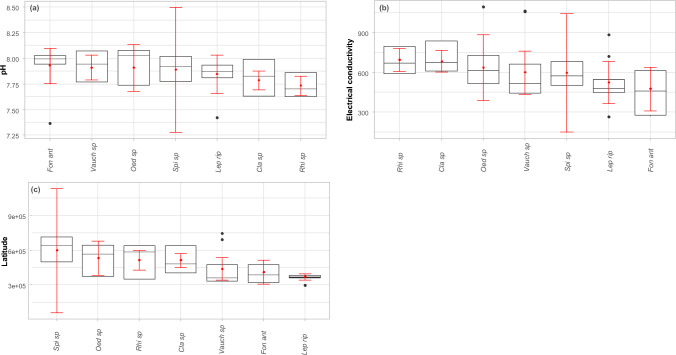
Boxplot reflecting the variability of the ecological parameters in rivers with non-vascular species. Only significant variables according to the Monte Carlo test and/or the BIOENV function are included: (**a**) pH, (**b**) Electrical conductivity, (**c**) Latitude Black is used to display the median, two hinges and two whiskers, and all “outlying” points individually. The red range gives the sample mean and lower and upper Gaussian confidence limits based on the t-distribution.

**Figure 2:**
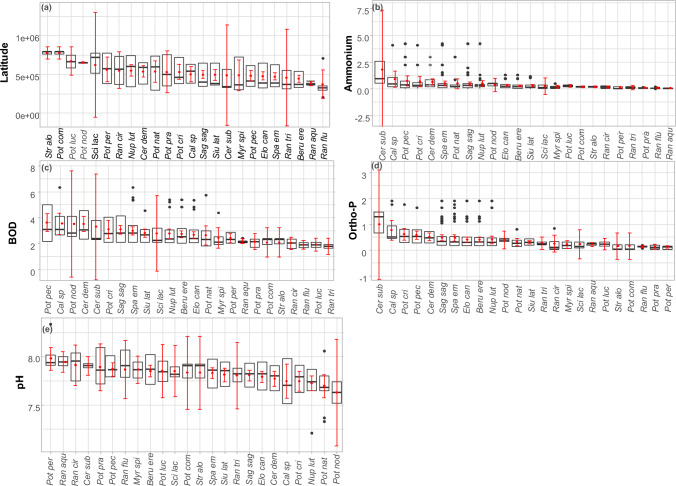
Boxplot reflecting the variability of the ecological parameters in rivers with vascular species. Only significant variables according to the Monte Carlo test and/or the BIOENV function are included: (**a**) Latitude, (**b**) Ammonium, (**c**) BOD, (**d**) Ortho-P, (**e**) pH. Black is used to display summary statistics (median, two hinges and two whiskers) and all “outlying” points individually. The red range gives the sample mean and lower and upper Gaussian confidence limits based on the t-distribution.

The range of environmental variables for each identified taxon is presented using boxplots separately for non-vascular taxa (Fig. 1) and vascular species (Fig. 2). Following significant variables according to the Monte Carlo test and/or BIOENV function were foud: pH, electrical conductivity and latitude for non-vascular taxa, and latitude, ammonium, BOD, orthophosphate and pH for vascular taxa.

Among the species of non-vascular macrophytes, *Spirogyra* sp. exhibited the greatest variation of distinguished significant parameters (Fig. 1). Distinguished in terms of ecological specificity are *Fontinalis antipyretica*, which develops in rivers with low electrical conductivity and high pH, and *Rhizoclonium* sp.and *Cladophora* sp*.*, which display preferences at the opposite extreme.

Among vascular macrophytes (Fig. 2) *Ceratophyllum submersum* exhibited the greatest range of ecological variability with respect to the significant variables (latitude, ammonium, BOD, ortho-P). There is also a distinguished group of five plant species that prefer more eutrophic waters (ammonium, ortho-P, BOD): *Ceratophyllum submersum*, *Ceratophyllum demersum*, *Callitriche* sp*.*, *Potamogeton pectinatus*, *Potamogeton crispus*.

### Multivariate analysis

**Figure 3:**
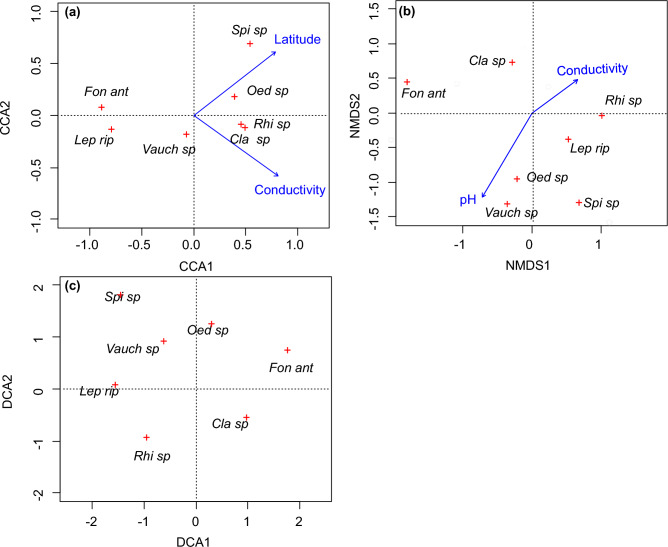
Ordination diagrams with seven non-vascular macrophyte scores plotted along the first two axes for: (**a**) CCA, (**b**) NMDS, (**c**) DCA. Taxon abbreviations are explained in Table 4.

The diagrams generated using the CCA and NMDS ordination methods facilitate the arrangement of species according to significant environmental gradients and visually represent the relative importance of a variable through the length of the corresponding arrow. In both cases, the direction of the arrow indicates the largest species turnover with respect to the variable, but the image of the ecological structure of the macrophyte community based on CCA (Fig. 3a) was different from that based on NMDS (Fig. 3b). First, in the case of CCA, the environmental vectors were arranged in relation to two largely independent directions, while in the case of NMDS, the environmental gradients represented a largely uniform direction. Moreover, these charts showed different ecological properties of individual species.

On the chart showing the results of the CCA analysis (Fig. 3a), the first axis explains 50.4% of the canonical variation, and the second axis only 21.6%. The significant environmental variables selected with the Monte Carlo test, electrical conductivity and latitude, exhibited a similar effect on the differentiation of non-vascular plants in rivers, as both were characterised by low p-values in the Monte Carlo test (Table 2) and a similar length of the vectors in the CCA diagram (Fig. 3a).

The results of the CCA analysis faithfully reflected the values shown in the univariate analyses, and the ordination of taxa with respect to electrical conductivity and latitude was uniform by both methods (Figs. 1 and 3a). The preferences of two extreme clusters with regard to these factors were confirmed by the difference in position between *Fontinalis antipyretica* and *Leptodictyum riparium* at one extreme, and *Rhizoclonium* sp*.* and *Cladophora* sp. at the other.

**Figure 4:**
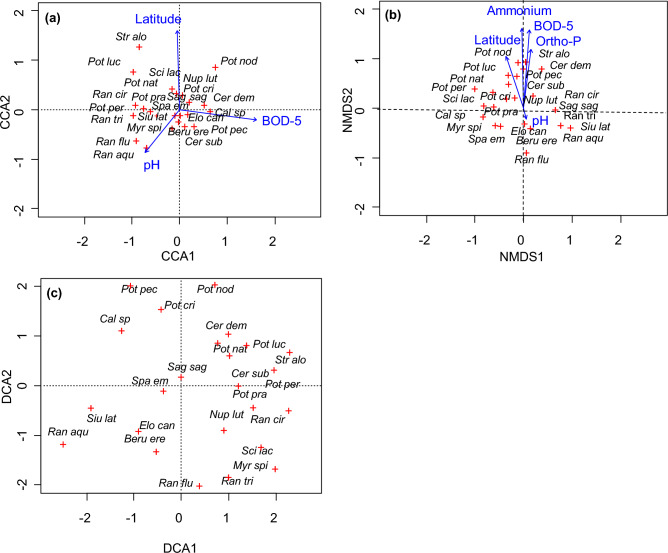
Ordination diagrams with vascular macrophyte scores plotted along the first two axes for (**a**) CCA, (**b**) NMDS, (**c**) DCA. Taxon abbreviations are explained in Table 4.

The results of the NMDS analysis are presented in the second diagram (Fig. 3b), which shows the ordination of non-vascular macrophytes with respect to the two variables selected in the BIOENV procedure: pH and electrical conductivity. Analyses based on metaMDS allowed us to obtain the best solution after 500 permutations, and the “stress” function was minimised to 0.07 (Table 4), which proves the stability of this solution. The results of the NMDS analysis showed a different community structure than in the case of CCA, and the demonstrated properties of individual species did not faithfully reflect the values shown in the univariate analyses (Fig. 1). While the highest mean electrical conductivity values were observed at the sites where *Cladophora* sp. (681.98) and *Rhizoclonium* sp. (693.60) were identified (Fig. 1), and the NMDS analysis also confirmed this (Fig. 3b), on the other hand, the extreme low electrical conductivity preference of *Fontinalis antipyretica* (473.9) was not visible. Similarly, ecological preferences for low pH were well identified for *Cladophora* sp. (7.78) and *Rhizoclonium* sp. (7.73) (Fig. 1), but the preference of *Fontinalis antipyretica* for acidic water (7.93) was not recognised in the NMDS analysis (Fig. 3b). The NMDS analysis also enabled determination of the significance of biological variables determined by permutation tests in fitted NMDS ordination (Fig. 4b). The species driving the site distribution pattern, referred to as intrinsic variables, can thus be identified.

In the DCA indirect gradient analysis, both stations and species are ordered in the space defined by the axes of the system (the first axis explains 30.7% of the variation and the second axis 24.9%) and the environmental gradient factors are hidden (Fig. 3c). The species ordination obtained based on the DCA analysis was very similar to that identified by NMDS, and was significantly different from the CCA ordination.

The ecological structures of vascular macrophyte communities based on the CCA and NMDS ordination methods differ as shown in the plots in Fig. 4a and b. In the case of CCA, the environmental vectors were arranged with respect to three independent directions, while NMDS arranged the environmental gradients almost unidirectionally. The graphs also show different properties of individual species of macrophytes.

The CCA analysis results (Fig. 4a), indicate that the first canonical axis explains 29.9% of the canonical variation, while the second canonical axis only 27.2%. The significant environmental variables selected based on the Monte Carlo test—pH, latitude, and BOD—had a similar effect on the differentiation of vascular plants, which was confirmed by similar very low p-values for each of the variables in the Monte Carlo test (Table 2) and similar lengths of the vectors on the CCA graph (Fig. 4a).

The CCA plot confirms to a large extent the ordination obtained by one-dimensional calculations (Fig. 2). For instance, CCA shows the similar position of the species *Stratiotes aloides*, *Potamogeton compressus*, *Potamogeton nodosus* and *Potamogeton lucens* (Fig. 4a) with respect to latitude. Similarly as in the one-dimensional analyses, the second cluster formed by the species *Ranunculus fluitans* and *Ranunculus aquatilis* indicated pH values higher than the average, and the species associated with eutrophic conditions—*Ceratophyllum demersum, Ceratophyllum submersum*, *Potamogeton pectinatus, Cladophora* sp*.* and *Potamogeton nodosus*—constituted a group with higher BOD values.

The NMDS analysis results for vascular plants are presented in the second diagram (Fig. 4b), which shows the species ordination for the variables selected in the BIOENV procedure, where the influence of ammonium and BOD was observed to be the greatest, and that of ortho-P and latitude was only slightly smaller. The ecological value of these variables is reflected by the length of the vectors in the diagram (Fig. 4b) and the corresponding p-values (Table 3). Analyses based on metaMDS allowed the best solution to be obtained after 500 tries, and the “stress” function was minimised to 0.14 (Table 3), proving this solution’s accuracy. The results of the NMDS analysis presented a different community structure than the CCA results, and the demonstrated properties of individual species did not always reflect the values shown in one-dimensional analyses. The NMDS diagram (Fig. 4b) only partly confirmed the results of one-dimensional analyses (Table 2) for the species *Potamogeton nodosus, Ceratophyllum demersum,* and *Potamogeton lucens*, which were found at sites with increased values of ammonium, BOD ortho-P and latitude, as well as for *Ranunculus fluitans*, growing in less eutrophic conditions. On the other hand, for several other species, NMDS ordination does not reflect the one-dimensional findings, for instance for *Stratiotes aloides*.

In the DCA analysis the first axis explained 31.4% of the variation, and the second axis 25.9% (Fig. 4c). The DCA analysis showed properties of the species similar to those given by NMDS. Compared with the CCA ordination, the DCA analysis displayed some differences.

NMDS analysis also enabled determination of the significance of biological variables identified by permutation tests in fitted NMDS ordination (Table 4). It is thus possible to find the group of species driving the site distribution pattern, referred to as intrinsic variables.

The levels of the “stress” parameter for non-vascular macrophytes (0.02) and for vascular macrophytes (0.143) indicate the good fit of the ordination model. With the exception of *Spirogyra* sp. in the non-vascular group, the presence of all taxa was associated to environmental factors. Within the vascular group, several species, including *Potamogeton crispus, Potamogeton pectinatus*, *Potamogeton perfoliatus*, *Berula erecta*, *Callitriche* sp., *Ceratophyllum demersum, Sium latifolium, Elodea canadensis* and *Sparganium emersum* demonstrated a strong response to environmental factors (Table 5).

**Table 5:** Significance of biological variables—the macrophytes which may drive the site distribution pattern, referred to as intrinsic variables, determined by permutation tests in fitted NMDS ordination (NMDS).

Taxa	R^2^	*p*-value	Stress	Taxa	R^2^	*p*-value	Stress
Non-vascular macrophytes				*Nup lut*	0.0466	0.116	0.143
* Cla sp*	0.6692	**0.001**	0.020	*Pot com*	0.0378	0.168	
* Oed sp*	0.1360	**0.024**		*Pot cri*	0.1196	**0.001**	
* Rhi sp*	0.5301	**0.001**		*Pot luc*	0.0394	0.130	
* Spi sp*	0.0833	0.095		*Pot nat*	0.0792	**0.022**	
* Vauch sp*	0.5008	**0.001**		*Pot nod*	0.0399	0.146	
* Fon ant*	0.3632	**0.001**		*Pot pec*	0.3330	**0.001**	
* Lep rip*	0.1185	**0.043**		*Pot per*	0.1254	**0.001**	
Vascular macrophytes				*Pot pra*	0.0209	0.381	
* Beru ere*	0.1755	**0.001**	0.143	*Ran aqu*	0.0509	0.074	
* Cal sp*	0.0785	**0.021**		*Ran cir*	0.0122	0.547	
* Cer dem*	0.3279	**0.001**		*Sci lac*	0.0172	0.385	
* Cer sub*	0.0179	0.433		*Siu lat*	0.2510	**0.001**	
* Elo can*	0.1393	**0.001**		*Spa em*	0.4122	**0.001**	
* Myr spi*	0.0313	0.227		*Str alo*	0.0378	0.168	

Significant values are in bold.

## Materials and methods

### Survey data

**Figure 5:**
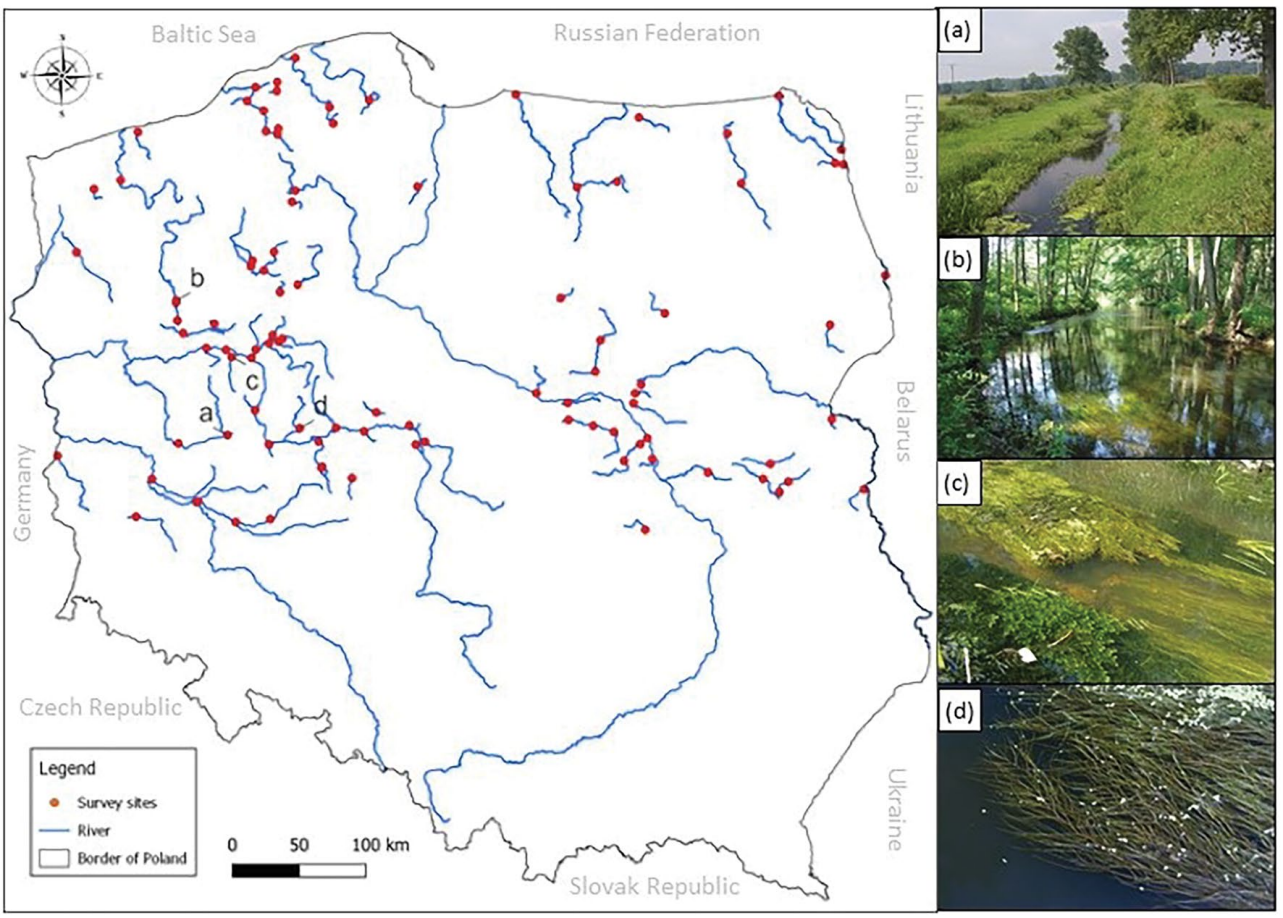
Location of selected survey sites. (**a**) Research station on the Mogilnica River, (**b**) Research station on the Płociczna River, (**c**) *Ceratophyllum demersum* L. s. s., *Potamogeton pectinatus* L., *Elodea canadensis* Michx, (**d**) *Potamogeton pectinatus* L.

The macrophyte survey was carried out at 100 river sites in the lowland area of Poland (Fig. 5).

To reduce habitat variability, a uniform river type was selected: small and medium lowland rivers with a sandy substrate, which are frequently distributed in Europe. Geologically, this is a siliceous river type, with substrate mainly consisting of fine-grained sand and sometimes clays or loesses, situated at elevations below 200 m above sea level. The catchment areas were smaller than 1000 km^2^. More characteristics of the survey sites can be found in a previous article^12^.

The research was conducted on 85 rivers. Of these, three were monitored at two sites each, eleven were monitored at two sites each, and the remaining rivers were monitored at one site each. Each of the selected survey sites was included in the national monitoring carried out between 2010 and 2013. For each site, ten environmental variables were obtained: latitude, longitude, ammonium nitrogen (abbreviation: ammonium), nitrate nitrogen (nitrate), organic nitrogen (organic-N), orthophosphate (ortho-P), organic phosphorous (organic-P), biological oxygen demand after five days (BOD), pH, and electrical conductivity. The chemical parameters were assessed based on 12 monthly measurements over one year. The macrophyte survey was carried out on a 100 m long river stretch, recording plants with submerged forms and floating leaves, whereas plants with only emergent forms were not included. Both, vascular plants (seed-bearing plants and ferns) as well as nonvascular mosses and filamentous algae (at the site, the occurrence or absence was recorded—incidental data). A glass-bottomed bucket was used to aid observations. The taxa identification based on^36,37^.

### Statistical analysis

#### Univariate analysis

The environmental data set, including ten variables collected for each survey site, was subjected to a series of descriptive analyses separately for non-vascular and vascular taxa. These characteristics included position measures (arithmetic mean, median), variation measures (standard deviation, coefficient of variation, range) and asymmetry measures (skewness coefficient). The range of environmental variables detected for each identified taxon was presented using boxplots^38^.

The descriptive analyses included only significant environmental variables, and the significance was verified according to the Monte Carlo test and/or the BIOENV function. The Monte Carlo test, an analysis of the influence of habitat features on the plant community ordering model, was performed^39,40^ based on 499 permutations, distinguishing the set of significant environmental variables (p < 0.05). The Monte Carlo selected variables were utilised for the CCA analysis. Based on the BIOENV function^29^ the best subset of environmental variables was found, and this was utilised for the NMDS analysis.

#### Multivariate analysis

In the next stage, three multivariate ordination techniques were applied: CCA, NMDS and DCA, of which CCA and DCA are based on a mutual averaging algorithm while NMDS is based on ranks.

The CCA analysis consisted in constructing a model of the relationship between the previously selected significant environmental parameters (based on the Monte Carlo test) and the biotic matrices, which were measured by the sum of squared deviations from the mean^39,40^. CCA results are presented as biplots, separately for each macrophyte group.

The NMDS analysis was based on the macrophyte data matrix, in which the Jaccard dissimilarity index was used to determine the similarity between the sites^41^. In addition, an approach was used^34^ which allows one to study the relationship between differences in the community structure due to differences in environmental variables—the results of botanical studies were compared with respect to environmental variables selected using the BIOENV procedure^29^. Based on BIOENV, the best subset of environmental variables among the ten considered was selected, maximising the rank correlation between the biotic similarity matrices of a specific macrophyte group and the environmental matrix so that the Euclidean distances of the scaled environmental variables had a maximum rank correlation with different species of organisms. The results were illustrated on biplots on which species data and selected environmental variables were presented. The metaMDS function was used to present the ordinance, where the number of dimensions was k = 2, and the minimum and maximum numbers of random starts in the search for a stable solution were taken to be 50 and 200, respectively.

Finally, DCA was used, an indirect gradient analysis method taking into account only the macrophyte data matrix for analyses^31^. As with the previous ordination techniques, the results were presented on a diagram.

The analyses were performed following the procedure implemented in the R 4.2.2 environment^42^. The BIOENV analysis and the permutation procedure were conducted using the BIOENV functions available in the vegan package^43^. The results of multivariate analyses were presented on biplots, on which the scaling was selected to optimise the spread of species in the area of the illustration so as to enable optimal assessment of the community structure and the environmental preferences of every taxon.

## Discussion

This study represents a significant effort that challenge the problem of interpreting various types of multidimensional ecological analyses based on real hydrobiological research findings. The research involved extensive field investigations covering a vast expanse of 100 river sites in the lowland area of Poland. These sites were carefully selected to ensure robust biological assessments and were supported by comprehensive environmental studies. Notably, monthly chemical analyses were conducted throughout the year, a practice that is relatively infrequent on that scale of biological studies.

To enhance the precision of the analyses, habitat variability was reduced by focusing on a uniform river type: small and medium lowland rivers with a sandy substrate. By doing so, the ordering of macrophytes with respect to the chemical and geographical variables taken into account in the analyses was not disturbed by other directions of variability such as geology (restricted to siliceous regions), sediment material (fine-grained sand, occasionally clays or loesses) and catchment area (smaller than 1000 km^2^). Moreover, the chosen river type that is widely distributed in Europe, making the research findings highly applicable across different contexts^11,12,44^.

Relying solely on a single statistical analysis in studying the ecological dynamics of macrophytes could result in a very limited understanding of their preferences in a diverse (multidimensional) environment. The consequences of such a narrow approach could be significant. It might lead to an oversimplified view of how macrophytes interact with their environment, potentially misinforming management and conservation strategies. For example, failing to recognize the influence of certain environmental gradients or stressors might lead to inappropriate recommendations for habitat restoration or species conservation. Thus, utilizing a broader array of statistical tools is essential to gain a comprehensive understanding of macrophytes' ecological dynamics in their naturally varied and complex habitats.

The three different ordination techniques used indicated slightly different roles of individual ecological factors in the differentiation of macrophytes in rivers, as well as different images of the species structure of plant communities. All the applied ordination techniques assume that species representing sites with similar environmental conditions are close to each other on the charts, while sites with more diverse characteristics should be further separated. Nevertheless, differences in the ordination results were expected to some extent, as these methods are based on radically different principles: a linear correlation model in CCA^30,32^ and the rank relationship between the similarities of individual positions in NMDS^29,45^. Multidimensional-rescaling ordinations are improved by DCA^31,35^. In contrast to traditional methods, based on comparative analyses of the obtained data with patterns adopted in guides and instructions for habitat mapping, the CCA and DCA methods based on the mutual averaging algorithm enable the ordering of surface characteristics in a multidimensional space^30^.

The CCA biplot, for both non-vascular and vascular species, showed the differentiated environmental preferences of individual species. The image was clearly revealed due to the inclusion of a small number of variables in the CCA analysis, which were limited to significant factors according to the Monte Carlo permutation test—there were two factors for non-vascular communities (electrical conductivity and latitude) and three for vascular communities (BOD, latitude, and pH). The risk of error is reduced by limiting the analysis to significant variables, since irrelevant environmental variables can distort the representation of gradients in a community structure in CCA analysis^46^.

The comparisons of the ecological preferences of individual species based on the CCA results and the basic descriptive statistics were broadly consistent. The strong convergence between CCA analyses and other descriptive statistics is a result of the fact that CCA is a direct gradient analysis method, where the relationship with the environmental variables is based on the linear correlation model^30,32^, which was confirmed in both analysed groups of macrophytes. Numerous studies have confirmed that CCA makes it possible to draw conclusions about the role of environmental variables in maintaining habitats for macrophytes^21,47^ and other groups of aquatic organisms^48,49^.

When analysing non-vascular plants in our database, we observed the indicated preferences for the water parameters, specifically electrical conductivity, consistently aligned with the position of the CCA vector and the calculated mean value. This consistency was evident for species related both positively to this gradient (*Rhizoclonium* sp., *Cladophora *sp.) as well as those that displayed negative relationship (*Fontinalis antipyretica*), considering the position of the CCA vector and the calculated mean value. The indicated preferences of these species are also consistent with those reported in the literature, where *Rhizoclonium *sp., *Cladophora *sp. are regarded as tolerant to water degradation, whereas *Fontinalis antipyretica* prefers less polluted rivers^23,25^.

In the case of vascular plants, the preferences for thelimnological variables, namely BOD, were also largely consistent both among positively related species (*Ceratophyllum demersum, Ceratophyllum submersum*, *Potamogeton pectinatus*, *Cladophora sp.*, *Potamogeton nodosus*) as well as those that displayed a negative relationship (e.g. *Potamogeton lucens*, *Stratiotes aloides*), as shown by CCA analysis and the calculation of means and medians. These preferences are also consistent with the literature where *Rhizoclonium *sp., *Cladophora *sp. are regarded as tolerant to water trophy and organic matter, whereas *Fontinalis antipyretica* is prefers less polluted rivers^23,25^. It was confirmed that eutrophication caused naturally or by anthropogenic activities can be a threat to various vascular plants.

The NMDS biplot provides a different image of the macrophyte community structure compared to the CCA method, yet it shares similarities with the DCA results. The NMDS method effectively defines the biological similarity of the relevant samples (not based on correlation) in terms of species composition, but maintains the rank relationship between these similarities in the distribution of samples, as outlined by Clarke^29^ and Dexter et al.^45^. Therefore, NMDS analysis proves to be a powerful tool for investigating multivariate relationships, especially when the data do not conform to the assumptions of multivariate normality. A similar approach is presented by Souza et al.^50^.

The analysis of the NMDS biplot focused on examining the correspondence between species and environmental vectors. In the case of vascular species, the environmental gradients exhibited a generally similar direction, making it challenging to discern the specific environmental preferences of individual species. Moreover, comparisons between the ecological preferences of certain species based on NMDS results and basic descriptive statistics yielded different outcomes. Regarding non-vascular plants, the preferences for the most critical factor, namely pH, were confirmed for some positively related plants (*Oedogonium *sp., *Vaucheria *sp. and *Fontinalis antipyretica*), but*,* the identification of preferences for *Spirogyra *sp. was inaccurate. Among the vascular plants, the positions on the NMDS diagram of *Potamogeton nodosus, Ceratophyllum demersum, Potamogeton lucens* confirmed their good development in eutrophic conditions as indicated by one-dimensional analysis and the literature^23,25^. Moreover, the position of *Ranunculus fluitans* with respect to trophy as indicated by the skewed ammonium, BOD and ortho-P vectors showed its relatively limited tolerance as determined by one-dimensional analysis and other sources^23,25^. It should be noted that the species ecological preferences, as indicated by the NMDS results, did not always align accurately with values obtained from one-dimensional analyses and CCA.

Detrended correspondence analysis is a multivariate statistical technique widely used by ecologists to identify the main factors or gradients in large, species-rich, but usually sparse data matrices that typify ecological community data. DCA is frequently used to suppress artefacts inherent in most other multivariate analyses. When applied to gradient data, DCA^31^, using a mutual averaging algorithm, is frequently used, among others, in organising positions^51^. The DCA biplot provides an alternative image of the macrophyte community structure, exhibiting much similarities with NMDS results and different from those obtained through CCA. DCA, like NMDS, is an indirect gradient analysis technique, while CCA is a direct gradient analysis method that relies on linear correlations to assess the species–environment relationship. relies. Accordingly, each method has its advantages, but also some weaknesses, therefore, it is advisable to take these into account when interpreting them when analyzing your ecological data.

The study used two analytical tools to identify the significant environmental factors: the Monte Carlo test, utilised in CCA, and the BIOENV test, developed for NMDS. These tools led to partially different identifications of the factors responsible for the development of macrophytes in rivers. This is because we are dealing with different types of analyses to test the significance of the variance explained by environmental factors.

The study was aimed at solving a problem in the field of analytical methodology, the research also uncovered ecological distinctions between vascular macrophytes and non-vascular bryophytes and algae were revealed. Significant factors influencing the development of aquatic plants were slightly different according to Monte Carlo and BIOENV. Nevertheless, for non-vascular plants, the importance of electrical conductivity was confirmed by both approaches. In contrast, vascular macrophytes were found to be sensitive to eutrophication, indicated by BOD (both methods), as well as to ammonia and phosphate (BIOENV). The importance of pH was confirmed for both macrophyte groups. The identification of groups of organisms representing different ecosystem functions is believed to be the key to understanding ecosystem processes and their response to environmental stress or disturbances^6,52,53^.

The research evaluated various ways of analysing ecological data that can be applied to aquatic organisms. Freshwater ecosystems are particularly vulnerable to degradation, and their environmental value and the benefits we derive from their uninterrupted functioning and rational use are great^54,55^. Accurate understanding of the requirements of aquatic species or macrophyte groups is essential for formulating appropriate measures for macrophyte conservation and for the preservation of fluvial habitats^26,27^. Moreover, due to the environmental sensitivity of macrophytes and their importance in biomonitoring, precision and caution in interpreting the results of mathematical analyses and modelling are recommended^12,47^. In solving these problems, advanced analytical techniques are very much needed, and in recent years multidimensional methods have proven extremely useful, although soon it will be necessary to face the challenge of interpreting new methods based on artificial intelligence^56,57^.

## Conclusions

Various analytical tools for determining the importance of ecological factors (Monte Carlo test, BIOENV) identify variable significant factors responsible for the macrophyte development in rivers.

A considerable similarity was found in the structures of macrophyte communities revealed by The NMDS and DCA methods, both indirect gradient analysis approaches reveal a significant similarity in the structures of macrophyte communities, while CCA, a direct gradient analysis method, results in a distinct ordering of macrophytes.

The CCA ordering reflects well the ecological preferences of aquatic plants as demonstrated by one-dimensional analyses. The ecological properties demonstrated by NMDS did not always accurately reflect the values given by one-dimensional analyses.

The NMDS analysis enabled determination of the significance of biological variables identified by permutation tests in fitted NMDS ordination, which makes it possible to identify the group of species driving the site distribution pattern.

The development of various groups of macrophytes is conditioned by different environmental drivers, and ecological differences between vascular macrophytes and non-vascular bryophytes and algae were revealed. The importance of electrical conductivity for non-vascular plants was confirmed, whereas vascular plants displayed sensitivity to eutrophication. The importance of pH was confirmed for both macrophyte groups.

## Data Availability

All data included in this study are available upon request by contact with the corresponding author.
